# A Regev-Type Fully Homomorphic Encryption Scheme Using Modulus Switching

**DOI:** 10.1155/2014/983862

**Published:** 2014-06-25

**Authors:** Zhigang Chen, Jian Wang, Liqun Chen, Xinxia Song

**Affiliations:** ^1^College of Computer Science and Technology, Nanjing University of Aeronautics and Astronautics, Nanjing 210016, China; ^2^College of Computer and Information, Zhejiang Wanli University, Ningbo, Zhejiang 315100, China; ^3^Information Security Group, Royal Holloway, University of London, Egham, Surrey TW20 0EX, UK; ^4^HP Labs, Bristol BS34 8QZ, UK; ^5^College of Junior, Zhejiang Wanli University, Ningbo, Zhejiang 315101, China

## Abstract

A critical challenge in a fully homomorphic encryption (FHE) scheme is to manage noise. Modulus switching technique is currently the most efficient noise management technique. When using the modulus switching technique to design and implement a FHE scheme, how to choose concrete parameters is an important step, but to our best knowledge, this step has drawn very little attention to the existing FHE researches in the literature. The contributions of this paper are twofold. On one hand, we propose a function of the lower bound of dimension value in the switching techniques depending on the LWE specific security levels. On the other hand, as a case study, we modify the Brakerski FHE scheme (in Crypto 2012) by using the modulus switching technique. We recommend concrete parameter values of our proposed scheme and provide security analysis. Our result shows that the modified FHE scheme is more efficient than the original Brakerski scheme in the same security level.

## 1. Introduction

A fully homomorphic encryption (FHE) scheme allows arbitrary functions on certain data (referred to as plaintexts) to be performed via their ciphertexts (the encrypted version of the plaintexts) without decrypting the ciphertexts first; therefore, performing these functions does not require one to hold the secret decryption key corresponding to the encryption algorithm. This cryptographic primitive has shown a variety of attractive applications both in theory and in practice. A typical application example is to outsource a computational job to a mistrusted remote server without compromising data privacy.

Since Gentry constructed the first FHE scheme in 2009 [1], a number of FHE schemes including various optimizations of the Gentry original scheme have been proposed. Gentry and colleagues developed several FHE schemes with different improvement, for example, [2–6]; one of them is how to bootstrap “packed” ciphertexts [6]. Smart and Vercauteren modified the Gentry scheme with the purpose of reducing the key and ciphertext sizes [7]. Stehlé and Steinfeld provides two improvements, respectively, on more aggressive analysis and probabilistic decryption algorithm in order to make the Gentry type of FHE schemes faster [8]. Brakerski et al. made a number of important contributions to this research field, such as [9–13], the details of which will be discussed more in the late part of this paper. Furthermore, van Dijk et al. proposed a new FHE construction over the integers [14], and Coron et al. further suggested on how to optimize this idea with shorter keys [15, 16]. López-Alt et al. constructed a multikey FHE scheme, which allows multiple ciphertexts under different keys to be decrypted jointly [17]. Alperin-Sheriff and Peikert introduced a method to achieve practical bootstrapping in Quasilinear time [18].

One critical challenge when constructing a FHE scheme is managing the noise growth in the process of homomorphic additions and multiplications. To our best knowledge, so far, there exist three techniques to manage the noise growth as follows.

The first technique is bootstrapping that was used in the first FHE scheme introduced by Gentry. Bootstrapping means to evaluate its own decryption circuit homomorphically. One can use a bootstrapping process to get a new ciphertext after each homomorphic addition or homomorphic multiplication. The noise level in the new ciphertext is maintained in a fixed level. As long as this noise level permits, one can handle the next homomorphic addition or multiplication. By recursing this process a leveled FHE scheme can be developed, and the number of levels (although say the depth of the levels) for a computational circuit could be arbitrary with an assumption of circular security. A FHE scheme with the property of having an arbitrary depth of leveled circuits is referred to as a “pure” FHE scheme.

The second technique is modulus switching. This technique was developed by Brakerski and Vaikuntanathan in [10] and improved in [11]. The main idea of modulus switching is to scale down the ciphertext vector **c** over *Z*_*q*_ or a factor *B* after each multiplication, which results in a new ciphertext vector **c**/*B* over *Z*_*q*/*B*_. A scaling process switches the first modulus *q* to the second modulus *q*/*B* and also reduces the noise *E* in the ciphertext vector **c** to the new noise *E*/*B* in the new ciphertext vector **c**/*B*. By following this process, the absolute magnitude of the new noise in the new ciphertext actually decreases. Modulus switching therefore can be used to manage noise at the cost of sacrificing the size of modulus. A leveled FHE scheme without bootstrapping can be achieved by modulus switching. In this technique, the depth of leveled computational circuits is prearranged before the computation starts. The depth is presented as a polynomial. For any prearranged polynomial denoted by *L*, one can evaluate circuits of depth *L* by carefully choosing the ladder of decreasing modulus.

The third technique is called Flatten, developed by Gentry et al. in [19]. It is designed for the case that an encryption key is presented as a vector and a ciphertext is presented as a matrix. It makes the coefficients of a vector or matrix small by using a flattening technique.

Among the three techniques for noise management, bootstrapping is a general technique that can be used to manage noise in any FHE scheme, but it is very costly! The technique of Flatten is only used in the case where ciphertexts are matrices and the secret keys are vectors. Modulus switching is a lightweight and very powerful way to manage noise and one can efficiently evaluate an arithmetic circuit with an arbitrary polynomial size without resorting to bootstrapping. In this paper, we will focus on modulus switching for noise management and consider the case that ciphertexts and the secret keys are both vectors.

In terms of noise growth, the noise grows from *E* to *E* ^2^ with every multiplication in most of the existing FHE schemes, where *E* denotes the noise magnitude in ciphertext. However, in the FHE scheme [12] by Brakerski in 2012 (we call it Bra12 for short), each homomorphic multiplication does not square the noise, and instead of the noise grows from *E* to* poly*(*n*) · *E* after each homomorphic multiplication. From this point of view, it looks like that the Bra12 scheme is more efficient, but in fact it is not true. Since the Bra12 scheme makes use of bootstrapping to manage noise, it requires modulus *q* must be big in order to achieve the result that the scheme has a circuit with enough depth to evaluate its own decryption circuit, for example, $q=\tilde{O}(2^{n/2})$. The security of the scheme depends on the ratio *q*/*B*, where *B* is an initial magnitude of noise; therefore, *B* cannot be small. These reasons result in the secret key **s** sampled uniformly from *Z*_*q*_^*n*^ rather than from the error distribution *χ* in the Bra12 scheme. In addition, the noise mainly depends on one norm of **s** writing as ||**s**||_1_ in homomorphic multiplication in the Bra12 scheme. In order to reduce the noise, the scheme uses binary decomposition of the secret key **s** to reduce the norm ||**s**||_1_. This means that a ciphertext **c** under the key **s** is converted into a new form of ciphertext, denoted by Powerof2(**c**) under key BitDecomp(**s**). Although the new form of the ciphertext and the secret key can effectively reduce the noise, it increases the dimension of ciphertext and the secret key. In particular, the dimension of the ciphertext and secret key can further blow up in homomorphic multiplication and key switching, which lead to a fatal result when evaluating deep circuits, since it may need too much memory to compute. This feature considerably affects efficiency in the Bra12 scheme.

In this paper, we use modulus switching and an additional technique to improve the efficiency of the Bra12 scheme. Our scheme has the following properties:

1. There is lower dimension of the ciphertext and the secret key in homomorphic multiplication and key switching than in the Bra12 scheme. The ciphertext for homomorphic multiplication is defined as ⌊2/*q*·(**c**_1_ ⊗ **c**_2_)⌉ that corresponds to the secret key **s** ⊗ **s** in our scheme, while the ciphertext for homomorphic multiplication is defined as ⌊2/*q* · (Poweof2(**c**_1_)⊗Powerof2(**c**_2_))⌉ that corresponds to the secret key BitDecomp(**s**) ⊗ BitDecomp(**s**) in the Bra12 scheme.
2. The secret key **s** is sampled from a Gaussian distribution *χ* in our scheme, which can enable us to get small coefficients of **s**. In the Bra12 scheme, the secret key **s** is sampled uniformly from *Z*_*q*_^*n*^.
3. Our scheme uses modulus switching to manage noise, while the Bra12 scheme uses bootstrapping to manage noise.
4. In our scheme the initial modulus is that *q* ≈ 2^*n*^*ε*^^ for every *ε* < 1, while in the Bra12 scheme the modulus is that $q\approx \tilde{O}(2^{n/2})$. The small modulus *q* makes our scheme considerably efficient.

For a FHE scheme using modulus switching, it is very important to choose a ladder of gradually decreasing moduli {*q*_*i*_}. However, so far there has not been a concrete method to tell how to choose these parameters in terms of a certain security level, even in the BGV scheme [11] that just provided a general method to choose moduli {*q*_*i*_}. In this paper, we provide a solution to this problem. We first derive a function between the lower bound on the dimension of the LWE problem and the security level. Then we can choose every concrete modulus *q*_*i*_ and other parameters for a certain security level (e.g., the security level is 80 bit) according to this function.

The rest of this paper is organized as follows.  Section 2 defines notational conventions, introduces the LWE assumption, and defines homomorphic encryption and its related terms.  Section 3 introduces the Regev encryption scheme that our scheme is based on and defines invariant structure. There is a minor change in the Regev encryption scheme that we describe here. We sample the secret key from a Gauss distribution rather than sample uniformly from *Z*_*q*_^*n*^ in the Regev encryption scheme.  Section 4 analyzes the homomorphic properties by the opinion of invariant structure and the noise growth in homomorphic addition and multiplication.  Section 5 introduces key switching and modulus switching. Our FHE scheme based on the modified Regev encryption scheme is presented in Section 6. We analyze how to enable the correctness of our scheme in Section 7. The security and the parameters of our scheme are presented in Section 8. We conclude the paper with a performance comparison between our scheme and the Bar12 scheme in Section 9.

## 2. Preliminaries

### 2.1. Basic Notation

For an integer *q*, we define the set *Z*_*q*_ = (−*q*/2, *q*/2]∩*Z*. For any *x* ∈ *Z*, let *y* = [*x*]_*q*_ denote the unique value *y* ∈ (−*q*/2, *q*/2]. We use ⌊*x*⌉ to indicate rounding *x* to the nearest integer, and ⌊*x*⌋, ⌈*x*⌉ (for *x*⩾0) to indicate rounding down or up. When *q* is not a power of two, we will use ⌈log⁡⁡*q*⌉ to denote 1 + ⌊log⁡*q*⌋.

We use *x* ← *D* to denote that *x* is a sample from a distribution *D*. We define *B*-bounded distributions as ones whose magnitudes never exceed *B*.

The inner product of two vectors **v**, **u** of dimension *n* is denoted by 〈**v**, **u**〉, recalling that 〈**v**, **u**〉 = **v** ^*T*^ · **u**. The tensor product of two vectors **v**, **u** of dimension *n*, denoted by **v** ⊗ **u**, is the *n* ^2^ dimensional vector containing all elements of the form **v**[*i*]**u**[*j*]. Note that 〈**v** ⊗ **u**, **x** ⊗ **y**〉 = 〈**v**, **x**〉 · 〈**u**, **y**〉.

A lattice is defined as the set of all integer combinations Λ = *L*(**b**_1_,…, **b**_*n*_) = {∑_*i*=1_^*n*^ *x*_*i*_ **b**_*i*_ : *x*_*i*_ ∈ *Z*  for  1 ≤ *i* ≤ *n*} of *n* linearly independent vectors **b**_1_,…, **b**_*n*_ in *R* ^*n*^. The set of vectors **b**_1_,…, **b**_*n*_ is called a basis for the lattice. A basis can be represented by the matrix **B** = [**b**_1_,…, **b**_*n*_] ∈ *R* ^*n*×*n*^. The determinant of a lattice is the absolute value of the determinant of the basis matrix det⁡⁡(Λ) = |det⁡⁡(**B**)|.

*q*-ary lattices are most important in lattice-based cryptography. Given a matrix **A** ∈ *Z*_*q*_^*n*×*m*^ for integers *q*, *m*, *n*, there are two kinds of *m*-dimensional *q*-ary lattices

(1) Λq(A)={y∈Zm:y=ATs mod⁡ q  for  some  s∈Zn},Λq⊥(A)={y∈Zm:ATs=0 mod⁡ q}.

The two kinds of *q*-ary lattices are dual to each other, namely, Λ_*q*_(**A**) = *q* · Λ_*q*_^⊥^(**A**)* and Λ_*q*_^⊥^(**A**) = *q* · Λ_*q*_(**A**)*.

### 2.2. Learning with Errors (LWE)

The learning with errors (LWE) problem was introduced by Regev [20]. This problem was later generalized as the ring learning with errors (RLWE) problem by Lyubashevsky et al. [21]. For security parameter *λ*, let *n* = *n*(*λ*) be an integer dimension, let *q* = *q*(*λ*)⩾2 be an integer, a vector **s** ∈ *Z*_**q**_^**n**^, and let *χ* = *χ*(*λ*) be a distribution over *Z*. Let *A*_**S**,*χ*_ be the distribution obtained by choosing a vector **a** from *Z*_*q*_^*n*^ uniformly at random and a noise term *e* ← *χ*, and outputting (**a**, 〈**a**, **s**〉 + *e*) ∈ *Z*_*q*_^*n*^ × *Z*_*q*_. The LWE problem includes the search-LWE problem and the decision-LWE problem. The search-LWE problem is giving an arbitrary number of independent samples from *A*_**S**,*χ*_, output **s** with a high probability. We are primarily interested in the decision-LWE (DLWE) problem for cryptographic applications. The DLWE problem is defined as follows.

**Definition 1 (DLWE).**

For an integer *q* = *q*(*λ*) and an error distribution *χ* = *χ*(*λ*) over *Z*, the decision-LWE problem, denoted by DLWE_*n*,*q*,*χ*_, is to distinguish the following two distributions: in the first distribution, one sample from *A*_**S**,*χ*_; in the second distribution, one sample uniformly from *Z*_*q*_^*n*+1^. The DLWE_*n*,*q*,*χ*_ assumption is that solving DLWE_*n*,*q*,*χ*_ is computationally infeasible.

Two kinds of reductions are known, namely, the quantum reduction [20] and classical [22, 23] reduction, between DLWE_*n*,*q*,*χ*_ and approximating short vector problems in lattices. Particularly, a probability distribution *χ* is taken to be the Gaussian distribution, which is statistically indistinguishable from the *B*-bound distribution for an appropriate value *B*.

Note that the DLWE problem can be seen as a bound distance decoding problem in *q*-ary lattices. The second component of LWE instance can be seen as a perturbed lattice point in Λ_*q*_(**A** ^*t*^), to be decoded.

We now state the quantum reduction from worst-case lattice problems to the LWE problem introduced in [20].

**Theorem 2 .**

For any integer dimension *n*, prime integer *q* = *q*(*λ*), and *B* = *B*(*λ*)⩾2*n*, there is an efficiently samplable *B*-bound distribution such that if there exists an efficient (possibly quantum) algorithm that solves *DLWE*_*n*,*q*,*χ*_, then there is an efficient quantum algorithm for solving $\tilde{O}(q\cdot n^{1.5}/B)$-approximate worst-case SIVP and gapSVP.

There are other forms of *q* (see [24, 25]). In addition, if the vector **s** is sampled from the distribution *χ*, then the LWE problem is still hard. We sample **s** from the Gaussian distribution *χ* in our scheme.

### 2.3. Leveled Fully Homomorphic Encryption

A homomorphic encryption scheme HE = (Keygen, Enc, Dec, Eval) includes a quadruple of PPT algorithms. For the definition of full homomorphic encryption, readers can refer to these papers [1, 12].

At present, there are two types of fully homomorphic encryption schemes. One is leveled fully homomorphic encryption schemes, in which the parameters of a scheme depend on the depth of the circuits that the scheme can evaluate. In that case any circuit with a polynomial depth can be evaluated. The other is pure fully homomorphic encryption schemes, which can be built from a leveled fully homomorphic encryption scheme with the assumption of circular security. A pure fully homomorphic encryption scheme can evaluate the circuit whose depth is not limited. The following definitions are taken from [12].

**Definition 3 (*L*-homomorphism).**

A scheme HE is *L*-homomorphic, for *L* = *L*(*λ*), if for any depth *L* arithmetic circuit *f* (over GF(2)) and any set of inputs, *m*_1_,…, *m*_*l*_, it holds that

(2) Pr[HE.Decsk(HE.Evalevk(f,c1,…,cl))≠f(m1,…,ml)] =negl(λ),

where (*pk*, *evk*, *sk*) ← HE.Keygen(1^*λ*^) and *c*_*i*_ ← HE.Enc_*pk*_(*m*_*i*_).

**Definition 4 (compactness, full homomorphism, and leveled full homomorphism).**

A homomorphic scheme is* compact* if its decryption circuit is independent of the evaluated function. A compact scheme is* fully homomorphic* if it is *L*-homomorphic for any polynomial *L*. The scheme is* leveled fully homomorphic* if it takes 1^*L*^ as additional input in key generation.

## 3. The Basic Encryption Scheme

As same as the Bra12 scheme, our scheme is based on Regev's encryption scheme [20]. We now describe the Regev encryption scheme, but we sample the secret key **s** from a Gauss distribution while it was sampled uniformly from *Z*_*q*_^*n*^ in the Regev encryption scheme. This modification allows us to achieve our goal that the error distribution *χ* can be set to be as small as possible in our scheme. We call this modified Regev encryption scheme the basic encryption scheme.

Let *n* = *n*(*λ*) be the dimension of lattice, an odd modulus *q* = *q*(*λ*), and an error distribution *χ* = *χ*(*λ*). The basic encryption scheme is described as follows.

  **E**.**SecretKeygen**(1^*λ*^)
: sample **s**′ ← *χ* ^*n*^. Output **s** **k** = **s** ← (1, **s**′).
 
**E**.**PublicKeygen**(**s**) : let *N*⩾2 (*n*log⁡⁡*q*). Sample **A**′ ← *Z*_*q*_^*N*×*n*^ and **e** ← *χ* ^*N*^. Compute **b** ← **A**′**s**′ + **e**. Set **A** to be the (*n* + 1)-column matrix consisting of **b** followed by the *n* columns of −**A**′, namely **A** = [**b**∣ − **A**′] ∈ *Z*_*q*_^*N*×(*n*+1)^. Note that **A** · **s** = **e**. Set the public key **p** **k** = **A**.
 
**E**.**Enc**(**pk**, *m*) : to encrypt a message *m* ∈ {0,1}, set **m** ← (*m*, 0,…, 0) ∈ {0,1}^*n*+1^, sample **r** ∈ {0,1}^*N*^, and output **c** ← ⌊*q*/2⌋ · **m** + **A** ^*T*^ · **r** ∈ *Z*_*q*_^*n*+1^.
 
**E**.**Dec**(**sk**, **c**) : output *m* ← ⌊(2/*q*)[〈**c**,**s**〉]_*q*_⌉mod⁡ 2.

The basic encryption scheme above is semantic security based on the hardness of the LWE problem. The proof of this statement follows the proof of security of the original Regev encryption scheme given in [20].

A FHE scheme needs to maintain an invariant structure in decryption that is composed of plaintext and noise. The scheme must keep the invariant structure in the process of homomorphic addition and homomorphic multiplication in order to achieve homomorphism. Next, we define the invariant structure in the above basic encryption scheme and explain the relationship between the correctness of decryption and the noise magnitude in ciphertext.

**Lemma 5 .**

Let **c** ∈ *Z*_*q*_^*n*+1^ and **s** ∈ *Z*_*q*_^*n*+1^ be two vectors such that

(3) 〈c,s〉=⌊q2⌋·m+e (mod⁡ q),

where *m* ∈ {0,1}. If |*e* | <⌊*q*/2⌋/2, then we have *m* ← **E**.**D** **e** **c**(**s**, **c**).

**Proof**

By definition

(4) 〈c,s〉=〈⌊q2⌋·m+AT·r,s〉 (mod⁡ q)=⌊q2⌋·m+rT·A·s (mod⁡ q)=⌊q2⌋·m+〈r,e〉 (mod⁡ q)=⌊q2⌋·m+e (mod⁡ q).

Since the coefficients of **e** are taken from a Gaussian distribution *χ*, *e* = 〈**r**, **e**〉 is also subject to a Gaussian distribution according to the standard fact from the Gaussian distribution. The Claim 5.2 in [20] showed that |*e*| < ⌊*q*/2⌋/2 with high probability. Consider an encryption of 0 now; it is closer to 0 than to ⌊*q*/2⌋ in this case and therefore the decryption is correct. The proof for an encryption of 1 is similar.

The term *e* is called the noise. ⌊*q*/2⌋ · *m* + *e* (mod⁡ *q*) is called the invariant structure. The above Lemma 5 shows that the invariant structure will be hold as long as |*e*| < ⌊*q*/2⌋/2, which can ensure the correctness of decryption. Note that it is very important to keep the invariant structure in ciphertexts generated in homomorphic evaluation.

## 4. Homomorphic Properties and Noise Analysis

We take the definition of homomorphic addition and homomorphic multiplication from the Bra12 scheme, but here we analyze the homomorphic properties of the above scheme by the approach of the invariant structure. Now we analyze the noise growth in the homomorphic addition and multiplication.

Let **c**_1_ and **c**_2_ be two ciphertexts under the same secret key **s** for modulus *q* such that

(5) 〈c1,s〉=⌊q2⌋·m1+e1(mod⁡ q)=⌊q2⌋·m1+e1+k1q,〈c2,s〉=⌊q2⌋·m2+e2(mod⁡ q)=⌊q2⌋·m2+e2+k2q,

for some *e*_1_ and *e*_2_.

### 4.1. Homomorphic Addition

Let **c** ^add^ = **c**_1_ + **c**_2_. If the invariant structure 〈**c**_1_ + **c**_2_, **s**〉 = ⌊*q*/2⌋ · (*m*_1_ + *m*_2_) + *e* (mod⁡ *q*) can be held during the decryption of **c** ^add^ for some *e*, the decryption would be correct such that homomorphic addition is obtained.

By definition

(6) 〈c1+c2,s〉=〈c1,s〉+〈c2,s〉=⌊q2⌋·(m1+m2) +e1+e2+k1q+k2q.

Let *e* ^add^ = *e*_1_ + *e*_2_. According to the Lemma 5, if |*e* ^add^| < ⌊*q*/2⌋/2, then *m*_1_ + *m*_2_ ← **E**.**D** **e** **c**(**s** **k**, **c**_1_ + **c**_2_). It also means that the invariant structure ⌊*q*/2⌋ · (*m*_1_ + *m*_2_) + *e*_1_ + *e*_2_ (mod⁡ *q*) can be kept in the decryption of **c** ^add^. We note that the noise term of output is the sum of input noises.

### 4.2. Homomorphic Multiplication

Multiplicative homomorphism cannot be straightforwardly achieved. We need to construct a form of the two input ciphertexts to represent the homomorphic multiplication such that we can get the product of the two plaintexts with respect to the input ciphertexts after decrypting the homomorphic multiplication. For this purpose, we now focus on the invariant structure in the process of decryption. If the invariant structure ⌊*q*/2⌋ · (*m*_1_ · *m*_2_) + *e* for some *e* is kept in the decryption of the homomorphic multiplication, we could achieve multiplicative homomorphism. Next, we describe how to achieve multiplicative homomorphism by the approach of the invariant structure.

Consider the multiplication of 〈**c**_1_, **s**〉 and 〈**c**_2_, **s**〉 now, we have:

(7) 〈c1,s〉·〈c2,s〉=(⌊q2⌋·m1+e1+k1q) ×(⌊q2⌋·m2+e2+k2q)=⌊q2⌋·⌊q2⌋·(m1m2)+⋯.

In order to keep the invariant structure ⌊*q*/2⌋ · (*m*_1_ · *m*_2_) + *e*, we multiple the above equation by 2/*q*:

(8) 2q·〈c1,s〉·〈c2,s〉 =⌊q2⌋·m1m2+m1e2+m2e1+2(e1k2+k1e2)  +q·(m1k2+k1m2+2k1k2)−[q]2·(m1k2+k1m2)  +[q]2q·(m1e2−m2e1−⌊q2⌋·(m1m2))+2q·e1e2 =⌊q2⌋·m1m2+e,

where *e* = *m*_1_ *e*_2_ + *m*_2_ *e*_1_ + 2(*e*_1_ *k*_2_ + *k*_1_ *e*_2_) + *q* · (*m*_1_ *k*_2_ + *k*_1_ *m*_2_ + 2*k*_1_ *k*_2_) − [*q*]_2_ · (*m*_1_ *k*_2_ + *k*_1_ *m*_2_) + [*q*]_2_/*q* ·(*m*_1_ *e*_2_ − *m*_2_ *e*_1_ − ⌊*q*/2⌋ · (*m*_1_ *m*_2_)) + 2/*q* · *e*_1_ *e*_2_.

The invariant structure appears in (8). Since 2/*q* · 〈**c**_1_, **s**〉 · 〈**c**_2_, **s**〉 = 〈2/*q* · (**c**_1_ ⊗ **c**_2_), **s** ⊗ **s**〉, multiplicative homomorphism is achieved by tensoring the input ciphertext 2/*q* · (**c**_1_ ⊗ **c**_2_). We note the ciphertext is fraction. For the sake of simplicity, we round the ciphertext for multiplication to the nearest integer ciphertext ⌊2/*q*·(**c**_1_ ⊗ **c**_2_)⌉, which will bring out an error **r** = ⌊2/*q*·(**c**_1_ ⊗ **c**_2_)⌉ − 2/*q* · (**c**_1_ ⊗ **c**_2_). Thus we get

(9) 〈⌊2q·(c1⊗c2)⌉,s⊗s〉 =〈2q·(c1⊗c2)+r,s⊗s〉 =〈2q·(c1⊗c2),s⊗s〉+〈r,s⊗s〉.

Plugging (8) into above equation, we have

(10) 〈⌊2q·(c1⊗c2)⌉,s⊗s〉 =⌊q2⌋·m1m2+m1e2+m2e1+2(e1k2+k1e2)  −[q]2·(m1k2+k1m2)+[q]2q  ·(m1e2−m2e1−⌊q2⌋·(m1m2))+2q·e1e2  +〈r,s⊗s〉 (mod⁡ q) =⌊q2⌋·m1m2+e1mult+e2mult(mod⁡ q),

where *e*_1_^mult^ = *m*_1_ *e*_2_ + *m*_2_ *e*_1_ + 2(*e*_1_ *k*_2_ + *k*_1_ *e*_2_) − [*q*]_2_ · (*m*_1_ *k*_2_ + *k*_1_ *m*_2_) + [*q*]_2_/*q* · (*m*_1_ *e*_2_ − *m*_2_ *e*_1_ − ⌊*q*/2⌋ · (*m*_1_ *m*_2_)) +2/*q* · *e*_1_ *e*_2_ and *e*_2_^mult^ = |〈**r**, **s** ⊗ **s**〉|. The noise is *e*_1_^mult^ + *e*_2_^mult^ in the ciphertext for multiplication. Particularly, the significant noise term of *e*_1_^mult^ is 2(*e*_1_ *k*_2_ + *k*_1_ *e*_2_), which is not like the many previous FHE schemes whose homomorphic multiplication operation squares the noise.

The ciphertext for multiplication can thus be defined as ⌊2/*q*·(**c**_1_ ⊗ **c**_2_)⌉ that can be decrypted using a tensored secret key **s** ⊗ **s**. The invariant structure in the decryption of the homomorphic multiplication is ⌊*q*/2⌋ · *m*_1_ *m*_2_ + *e*_1_^mult^ + *e*_2_^mult^. If |*e*_1_^mult^ + *e*_2_^mult^| < ⌊*q*/2⌋/2, according to Lemma 5, the invariant structure can be kept such that the correctness of decryption can hold. So we have *m*_1_ *m*_2_ ← **E**.**D** **e** **c**(**s** **k**, ⌊2/*q*·(**c**_1_ ⊗ **c**_2_)⌉), where **s** **k** is **s** ⊗ **s**. So far, we have finished the construction for the ciphertext for multiplication. We have achieved homomorphic addition and homomorphic multiplication. However, the noise growth is caused in the homomorphic addition and homomorphic multiplication.

The problem of noise growth in the homomorphic evaluation affects directly the homomorphic ability of the above basic encryption scheme, so it is critical to manage noise growth for constructing the FHE scheme. Before we solve the problem of noise growth, we in the next subsection analyze the noise growth in a homomorphic addition and homomorphic multiplication. Note that our analysis method for the noise growth is different from the one used in the Bra12 scheme, as the secret key **s** is sampled from a Gaussian distribution which results in the secret key **s** is *B*-bounded. In addition, we give a tighter noise analysis than it in [12].

### 4.3. Noise Analysis

**Lemma 6 .**

Let *q*, *n*, |*χ* | ⩽*B*, *N* be parameters as described in the basic encryption scheme. Let **c**_1_, **c**_2_ be the ciphertexts under the secret key **s** such that

(11) 〈c1,s〉=⌊q2⌋·m1+e1(mod⁡ q),〈c2,s〉=⌊q2⌋·m2+e2(mod⁡ q),

with |*e*_1_ | , |*e*_2_ | ⩽*E* < ⌊*q*/2⌋/2.Then

(12) 〈c1+c2,s〉=⌊q2⌋·[m1+m2]2+eadd(mod⁡ q),〈⌊2q·(c1⊗c2)⌉,s⊗s〉=⌊q2⌋·m1m2+emult(mod⁡ q),

where |*e* ^*add*^| ⩽ 1 + 2*E*, |*e* ^*mult*^| ⩽ 12*nB* *E*.

**Proof**

*Analysis for Addition.* By definition

(13) 〈c1+c2,s〉=〈c1,s〉+〈c2,s〉  (mod⁡ q)=⌊q2⌋·m1+e1+⌊q2⌋·m2+e2 (mod⁡ q)=⌊q2⌋·(m1+m2)+e1+e2 (mod⁡ q)=⌊q2⌋·[m1+m2]2+2·⌊q2⌋·⌊(m1+m2)2⌋ +e1+e2 (mod⁡ q).

Then we get |*e* ^add^| = |2 · ⌊*q*/2⌋ · ⌊(*m*_1_ + *m*_2_)/2⌋ + *e*_1_ + *e*_2_ | ⩽1 + 2 · *E*.

*Analysis for Multiplication.* By (10)

(14) 〈⌊2q·(c1⊗c2)⌉,s⊗s〉=⌊q2⌋·m1m2 +e1mult+e2mult(mod⁡ q).

We first analyze the bound of *e*_1_^mult^. The magnitude of *e*_1_^mult^ mainly depends on the term 2(*e*_1_ *k*_2_ + *k*_1_ *e*_2_), so we check the bound of the absolute value of *k*_1_ (the same bound also holds for *k*_2_):

(15) |k1|=|〈c1,s〉−⌊q/2⌋·m1−e1|q⩽|〈c1,s〉|q+1⩽(||c1||∞q)·||s||1+1⩽(12)·||s||1+1⩽nB.

The absolute value of *k*_1_ depends on ||**s**||_1_ from above inequality, then the bound of *k*_1_ is *O*(*E* · ||**s**||_1_). The tighter bound is described as follows:

(16) |e1mult|⩽2E+4nB E+2nB+3⩽8nB E.

Next, we analyze the bound of *e*_2_^mult^. According to the definition of an error **r** = ⌊2/*q*·(**c**_1_ ⊗ **c**_2_)⌉ − 2/*q* · (**c**_1_ ⊗ **c**_2_) and the secret key sampled from a *B*-bounded Gaussian distribution, we get ||**r**||_*∞*_ ⩽ 1/2 and |**s** ⊗ **s** | ⩽(*n*+1)^2^ *B* ^2^. Then

(17) |e2mult|=|〈r,s⊗s〉|⩽||r||∞·|s⊗s|⩽(n+1)2B2.

Putting these together, we get

(18) |e1mult+e2mult|⩽8nB E+(n+1)2B2⩽8nB E+4nB E⩽12nB E.

We see that the significant noise term in the homomorphic multiplication depends on ||**s**||_1_ from Lemma 6, which also happens in the Bra12 scheme. In order to reduce the norm, the secret key **s** is expressed in the form of binary, namely, BitDecomp(**s**), then the ciphertext corresponding to the **s** is expressed in Powerof2(**c**). The side effect is to produce the ciphertext vector and the secret key vector of a high dimension. In particularly, the ciphertext is the form of ⌊2/*q* · (*Powerof*2(**c**_1_) ⊗ *Powerof*2(**c**_2_))⌉ under the key BitDecomp(**s**_1_) ⊗ BitDecomp(**s**_2_) after homomorphic multiplication, which results in a large amount of computation that requires a large memory. The process cannot be practical. However, our scheme does not have this result. Since we sample the secret key from a Gaussian distribution that enables the coefficients of the secret key to be as small as possible, the secret key **s** needs not to be expressed in the form of binary, so the ciphertext. That is the reason why it can improve performance.

Under the above definition of homomorphic addition and homomorphic multiplication, we can perform only a bounded number of homomorphic operations (namely, a somewhat homomorphic encryption scheme), because the noise and the dimension grow as a result of performing homomorphic operations. Therefore, there are two problems that should be solved in order to achieve a FHE scheme based on the somewhat homomorphic encryption scheme.

First, we need to control the dimension of the ciphertext that increases from *n* + 1 to (*n*+1)^2^ after a homomorphic multiplication. We use the key switching technique to solve this problem.

Second, we need to manage the noise growth in homomorphic operations. We use modulus switching to solve this problem.

## 5. Key Switching and Modulus Switching

We describe the two techniques: key switching and modulus switching. Our notation is adopted from [11].

### 5.1. Key Switching

Key switching can transform a ciphertext **c**_1_ under a secret key **s**_1_ to a new ciphertext **c**_2_ under a secret key **s**_2_, in which **c**_1_ and **c**_2_ encrypt the same message. If the dimension of **c**_2_ and **s**_2_ is lower than the dimension of **c**_1_ and **s**_1_, the dimension of the key and ciphertect vectors is reduced by key switching.

Key switching consists of two procedures. The first procedure is denoted by **S** **w** **i** **t** **c** **h** **K** **e** **y** **G** **e** **n**(**s**_1_, **s**_2_, *n*_1_, *n*_2_, *q*), which takes as input the two secret key vectors, the respective dimension of these vectors, the corresponding modulus *q*, and outputs some auxiliary information *τ* that is a matrix. The second procedure is denoted by **S** **w** **i** **t** **c** **h** **K** **e** **y**(*τ*, **c**_1_, *n*_1_, *n*_2_, *q*), which takes as input the auxiliary information *τ*, a ciphertext **c**_1_, and its dimension *n*_1_, the dimension of the output ciphertext *n*_2_, and the modulus *q*, and outputs a new ciphertext **c**_2_ whose dimension is *n*_2_.

 
**S** **w** **i** **t** **c** **h** **K** **e** **y** **G** **e** **n**(**s**_1_ ∈ *Z*_*q*_^*n*_1_^, **s**_2_ ∈ *Z*_*q*_^*n*_2_^):
  1. Run **A** ← **E**.**P** **u** **b** **l** **i** **c** **K** **e** **y** **g** **e** **n**(**s**_2_) for *N* = *n*_1_ · ⌈log⁡⁡*q*⌉, namely, **A** = [**b**∣ − **A**′].
  2. Set **B** ← [(Powerof2(**s**_1_) + **b**)−**A**′], which means to add the Powerof2(**s**_1_) ∈ *Z*_*q*_^*N*^ to −**A**′ 's first column and add **b** to −**A**′ 's second column. Output *τ*_**s**_1_→**s**_2__ = **B**.
 
**S** **w** **i** **t** **c** **h** **K** **e** **y**(*τ*_**s**_1_→**s**_2__, **c**_1_, *q*): output **c**_2_ = BitDecomp(**c**_1_)^*T*^ · **B** ∈ *Z*_*q*_^*n*_2_^.

Key switching is essentially the product of a high dimension vector and a high dimension matrix. Next, we describe the correctness of key switching; namely, the decryption of the new ciphertext can preserve correctness. The proof is based on the definition (see [11]).

**Lemma 7 .**

Let **s**_1_, **s**_2_, *q*, **A**, **B** = *τ*_**s**_1_→**s**_2__ be parameters as described in **S** **w** **i** **t** **c** **h** **K** **e** **y** **G** **e** **n** and have **A** · **s**_2_ = **e**_2_ ∈ *Z*_*q*_^*N*^. Let **c**_1_ ∈ *Z*_*q*_^*N*^ and **c**_2_ ← **S** **w** **i** **t** **c** **h** **K** **e** **y**(*τ*_**s**_1_→**s**_2__, **c**_1_). Then,

(19) 〈c2,s2〉=〈BitDecomp⁡(c1),e2〉+〈c1,s1〉(mod⁡ q).

### 5.2. Modulus Switching

**Definition 8 (Scale).**

For integer vector **x** and integers *q* > *p* > *m*, we define **x**′ ← **S** **c** **a** **l** **e**(**x**, *q*, *p*, 2) to be the vector closest to (*q*/*p*) · **x** that satisfies **x** = **x**′(mod⁡ 2).

The next lemma shows that it is possible to transform a ciphertext **c** that encrypts *m* under key **s** for modulus *q* into a ciphertext **c**′ that encrypts *m* under the same key **s** for modulus *p*. Since our basic encryption scheme is different from the basic scheme in the BGV scheme [11], the proof of Lemma 9 is slightly different from the proof in [11].

**Lemma 9 .**

Let *q*, *p* be odd and *q* > *p* > 2. Let **c** ∈ *Z*_*q*_^*n*+1^ and **c**′ ← **S** **c** **a** **l** **e**(**x**, *q*, *p*, 2). Then, for any **s** ∈ *Z*_*q*_^*n*+1^, if 〈**c**, **s**〉 = ⌊*q*/2⌋ · *m* + *E* (mod⁡ *q*) and 〈**c**′, **s**〉 = ⌊*p*/2⌋ · *m* + *E*′(mod⁡ *p*), with |*E* | <*q*/4 − (*q*/*p*) · ||**s**||_1_ − *q*/(2*p*), we have

(20) |E′|<(pq)·|E|+||s||1+12,⌊2p(〈c′,s〉mod⁡ p)⌉=⌊2q〈c′,s〉mod⁡ q⌉mod⁡ 2.

**Proof**

By 〈**c**, **s**〉 = ⌊*q*/2⌋ · *m* + *E* (mod⁡ *q*), we have 〈**c**, **s**〉 − ⌊*q*/2⌋ · *m* − *kq* = *E* for some *k* ∈ *Z*. For the same *k*, let 〈**c**′, **s**〉 − ⌊*p*/2⌋ · *m* − *kp*. Next we just prove |〈**c**′, **s**〉 − ⌊*p*/2⌋ · *m* − *kp* | <*p*/2 in order to prove 〈**c**′, **s**〉 − ⌊*p*/2⌋ · *m* − *kp* = *E*′.

Since 〈**c**′, **s**〉 = 〈(*p*/*q*) · **c**, **s**〉 + 〈*ε*, **s**〉, where ||*ε*||_*∞*_ < 1, we have

(21) |〈c′,s〉−⌊q2⌋·m−kp| =|〈(pq)·c,s〉−⌊q2⌋·m−kp+〈ε,s〉| =|(pq)·⌊q2⌋·m+(pq)·E   +kp−⌊p2⌋·m−kp+〈ε,s〉| =|⌊p2⌋·m+(12−p2q)·m+(pq)·E   −⌊p2⌋·m+〈ε,s〉| =|(12−p2q)·m+(pq)·E+〈ε,s〉| <(pq)·|E|+||s||1+12 <p4.

We thus have 〈**c**′, **s**〉 − ⌊*p*/2⌋ · *m* − *kp* = *E*′ and |*E*′ | <(*p*/*q*) | *E* | +||**s**||_1_ + 1/2.

Since 〈**c**′, **s**〉 mod *p* = 〈**c**′, **s**〉 − *kp* and **c** = **c**′(mod 2), we have 〈**c**′, **s**〉 mod *p* = 〈**c**′, **s**〉 − *kp* = 〈**c**, **s**〉 − *kq*(mod 2) = 〈**c**, **s**〉 mod *q*(mod 2). By definition, 2*p* = 2*q*(mod 2). Since *pq* and 2 are coprime, it follows that 2/*q* = 2/*p*(mod 2). Modulo 2, we have (2/*p*)·(〈**c**′, **s**〉mod⁡ *p*) = (2/*q*)·(〈**c**, **s**〉mod⁡ *q*)(mod 2). We thus get ⌊(2/*p*)(〈**c**′, **s**〉mod⁡ *p*)⌉ = ⌊(2/*q*)(〈**c**, **s**〉mod⁡ *q*)⌉ mod 2.

The following corollary follows immediately from Lemma 9.

**Corollary 10 .**

Let *q* and *p* be two odd moduli. Let **c** be a ciphertext under the key **s** for the modulus *q*, where *m* ← ⌊(2/*q*)(〈**c**, **s**〉 mod *q*)⌉ mod 2. Suppose that **s** is a completely short key, and assume that |*E* | <*q*/4 − (*q*/*p*) · ||**s**||_1_ − *q*/(2*p*). Then we have **c**′ ← **S** **c** **a** **l** **e**(**x**, *q*, *p*, 2), where **c**′ is a ciphertext that encrypts the same message *m* under the key **s** for the modulus *p*, namely, *m* ← ⌊(2/*p*)(〈**c**′, **s**〉 mod *p*)⌉ mod 2. The noise of the new ciphertext **c**′ has magnitude at most (*p*/*q*)·|*E* | +||**s**||_1_ + 1/2.

Since the noise magnitude in the ciphertext **c** depends on the length of the key vector **s**, we must make the length of the key vector **s** short in order to use modulus switching to reduce the magnitude of the noise. For this purpose, we sample the key **s** from Gaussian distribution that is set to be as small as possible.

## 6. A Regev-Type FHE Scheme Using Modulus Switching

Next, we use modulus switching to construct a Regev-type FHE. This scheme is a leveled FHE scheme, in which the *i*th level needs a modulus *q*_*i*_. The parameters in our scheme includes a ladder of decreasing modulus {*q*_*i*_} (*i* = *L* − 1,…, 0), where a parameter *L* indicates the depth that a circuit can be evaluated. It is very important to choose reasonable modulus from *q*_*L*_ to *q*_0_, and we will focus on the details on how to choose reasonable modulus in Section 8. Since the magnitude of *q*_*L*_ is related to the security parameter *λ* and different circuit depths result in different magnitude values of *q*_*L*_, the performance of our scheme depends on the security parameter *λ* and the circuit depth *L*.

 
**F** **H** **E**.**S** **e** **t** **u** **p**(*λ*, *L*): input the security parameter *λ* and the circuit level *L*, output a ladder of decreasing modulus {*q*_*i*_} (*i* = *L* − 1,…, 0), the noise distribution *χ*, and the dimension *n*. Note that *χ* and *n* are the same as in the previous basic encryption scheme.
 
**F** **H** **E**.**K** **e** **y** **G** **e** **n**(*n*, *i*): For *i* = *L* − 1 down to 0, do the following.
  1. Run **s**_*i*_ ← **E**.**S** **e** **c** **r** **e** **t** **K** **e** **y** **g** **e** **n**(1^*n*^). Let **s** **k** = {**s**_*i*_}.
  2. Run **A**_*i*_ ← **E**.**P** **u** **b** **l** **i** **c** **K** **e** **y** **g** **e** **n**(**s**_*i*_). Let **p** **k**_1_ = {**A**_*i*_} (*i* = *L* − 1,…, 0).
  3. Set **s**_*i*_′ ← **s**_*i*_ ⊗ **s**_*i*_ ∈ *Z*_*q*_^(*n* + 1)^2^^.
  4. Run *τ*_**s**_*i*+1_′→**s**_*i*__ ← **S** **w** **i** **t** **c** **h** **K** **e** **y** **G** **e** **n**(**s**_*i*+1_′, **s**_*i*_). (Omit this step when *i* = *L* − 1.) Let **p** **k**_2_ = {*τ*_**s**_*i*+1_′→**s**_*i*__} (*i* = *L* − 1,…, 0).
 Then output **s** **k** = {**s**_*i*_} and **p** **k** = (**p** **k**_1_, **p** **k**_2_).
 
**F** **H** **E**.**E** **n** **c**(**p** **k**, *m*): take a message *m* ∈ {0,1}. Run **E**.**E** **n** **c**(**A**_*L*−1_, *m*).
 
**F** **H** **E**.**D** **e** **c**(**s** **k**, **c**_*i*_): assume that **c**_*i*_ is a ciphertext under the secret key **s**_*i*_. Run **E**.**D** **e** **c**(**s** **k**, **c**_*i*_).
 
**F** **H** **E**.**A** **d** **d**(**p** **k**, **c**_1_, **c**_2_): input two ciphertexts **c**_1_, **c**_2_ under the same secret key **s**_*i*_. If the two secret keys are different, we can use** FHE.Refresh **to refresh the two ciphertexts to the new two ciphertexts under the same secret key. Then, output **c**_3_ ← **c**_1_ + **c**_2_.
 
**F** **H** **E**.**M** **u** **l** **t**(**p** **k**, **c**_1_, **c**_2_): input two ciphertexts **c**_1_, **c**_2_ under the same secret key **s**_*i*_. If the two secret keys are different, we can use** FHE.Refresh **to make it so. Compute **c**_3_ ← ⌊2/*q* · (**c**_1_ ⊗ **c**_2_)⌉, and the relative secret key is **s**_*i*_′ = **s**_*i*_ ⊗ **s**_*i*_. Then, output **c**_4_ ← **F** **H** **E**.**R** **e** **f** **r** **e** **s** **h**(**c**_3_, *τ*_**s**_*i*+1_′→**s**_*i*__, *q*_*i*_, *q*_*i*−1_).
 
**F** **H** **E**.**R** **e** **f** **r** **e** **s** **h**(**c**, *τ*_**s**_*i*_′→**s**_*i*−1__, *q*_*i*_, *q*_*i*−1_): input ciphertext **c** under the secret key **s**_*i*_′ for modulus *q*_*i*_. *τ*_**s**_*i*_′→**s**_*i*−1__ is the auxiliary information for key switching. The current and next modulus are *q*_*i*_ and *q*_*i*−1_. Do the following.
  1. Key switching: comput **c**_1_ ← **S** **w** **i** **t** **c** **h** **K** **e** **y**(*τ*_**s**_*i*_′→**s**_*i*−1__, **c**, *q*_*i*_), a ciphertext under the key **s**_*i*−1_ for *q*_*i*_.
  2. Modulus switching: compute **c**_2_ ← **S** **c** **a** **l** **e**(**c**_1_, *q*_*i*_, *q*_*i*−1_, 2), a ciphertext under the key **s**_*i*−1_ for *q*_*i*−1_.

In order to enable the correctness of the above leveled FHE scheme, we must choose the correct parameters. Next, we describe how to enable the correctness of this scheme.

## 7. Correctness

The correctness of the above leveled FHE scheme comes from the correctness of each step in homomorphic operations, that is, each step in** FHE.Add** and** FHE.Mult**. If the noise magnitude in ciphertext is below *q*_*i*−1_/4 or *q*_*i*_/4 after each step in homomorphic operations, correct decryption is guaranteed.

### 7.1. The Initial Noise

The initial ciphertext is output by** FHE.Enc **that just invokes** E.Enc**.

**Lemma 11 .**

Let *q*_*L*−1_, *n*, *N* be the parameters associated with** FHE.Enc**. *χ* is a *B*-bounded Gaussian distribution. The length of the noise in ciphertexts output by** FHE.Enc** is at most *NB*. If *NB* < ⌊*q*_*L*−1_/2⌋/2, correct decryption is guaranteed.

**Proof**

**E**.**E** **n** **c**(**A**_*L*−1_, *m*) shows that ⌊*q*/2⌋ · **m** + **A**_*L*−1_^*T*^ · **r** ∈ *Z*_*q*_*L*−1__^(*n*+1)^, where **r** ∈ {0,1}^*N*^. We have **A**_*L*−1_ · **s**_*L*−1_ = **e**, where **e** ← *χ* ^*N*^ and **s**_*L*−1_ ← **F** **H** **E**.**K** **e** **y** **G** **e** **n**. Then we get

(22) 〈c,sL−1〉(mod⁡ qL−1) =⌊q2⌋·m+rTAL−1sL−1(mod⁡ qL−1) =⌊q2⌋·m+NB(mod⁡ qL−1).

According to Lemma 5, if *NB* < ⌊*q*_*L*−1_/2⌋/2, then correct decryption is guaranteed.

### 7.2. The Correctness of Homomorphic Operations

**Lemma 12 .**

Let **c**_1_ and **c**_2_ be two ciphertexts under **s**_*i*_ for *q*_*i*_, where 〈**c**_1_, **s**_*i*_〉 = ⌊*q*_*i*_/2⌋ · *m*_1_ + *e*_1_(mod⁡ *q*_*i*_) and 〈**c**_2_, **s**_*i*_〉 = ⌊*q*_*i*_/2⌋ · *m*_2_ + *e*_2_(mod⁡ *q*_*i*_) with |*e*_1_|, |*e*_2_| ⩽ *E* < ⌊*q*_*i*_/2⌋/2. Let **c**_3_ = **c**_1_ + **c**_2_. The noise magnitude of **c**_3_ is at most 2*E*. If 2*E* < ⌊*q*_*i*_/2⌋/2, we have *m*_1_ + *m*_2_ ← **F** **H** **E**.**D** **e** **c**(**s**_*i*_, **c**_3_); namely, **c**_3_ can be correctly decrypted.

**Proof**

The proof can be obtained easily from Lemma 6.

The procedure of** FHE.Mult** consists of three steps, namely, the multiplication, and then the key switching and modulus switching. Next, we analyze the correctness of each step.

**Lemma 13 .**

Let **c**_1_ and **c**_2_ be two ciphertexts under **s**_*i*_ for *q*_*i*_, where 〈**c**_1_, **s**_*i*_〉 = ⌊*q*_*i*_/2⌋ · *m*_1_ + *e*_1_(mod⁡  *q*_*i*_) and 〈**c**_2_, **s**_*i*_〉 = ⌊*q*_*i*_/2⌋ · *m*_2_ + *e*_2_(mod⁡  *q*_*i*_) with |*e*_1_|, |*e*_2_| ⩽ *E* < ⌊*q*_*i*_/2⌋/2. Let **c**_3_ = ⌊2/*q* · (**c**_1_ ⊗ **c**_2_)⌉, and let **s**_*i*_′ = **s**_*i*_ ⊗ **s**_*i*_. The noise magnitude of **c**_3_ is at most 12*nB* *E*. If 12*nB* *E*<⌊*q*_*i*_/2⌋/2, we have *m*_1_ *m*_2_ ← **F** **H** **E**.**D** **e** **c**(**s**_*i*_′, **c**_3_); that is, **c**_3_ can be correctly decrypted.

**Proof**

The proof can be obtained easily from Lemma 6.

We note that the noise after multiplication is 12*nB* *E* rather than *E* ^2^ like in many of the previous FHE schemes.

**Lemma 14 .**

Let **c** be a ciphertext under **s**_*i*_′ = **s**_*i*_ ⊗ **s**_*i*_ for *q*_*i*_, where 〈**c**, **s**_*i*_′〉 = ⌊*q*_*i*_/2⌋ · *m* + *e* (mod⁡ *q*_*i*_) with |*e*| ⩽ *E* < ⌊*q*_*i*_/2⌋/2. Let **c**_1_ ← **S** **w** **i** **t** **c** **h** **K** **e** **y**(*τ*_**s**_*i*_′→**s**_*i*−1__, **c**, *q*_*i*_). The noise magnitude of *c*_1_ is at most *E* + *B*(*n*+1)^2^log⁡⁡*q*_*i*_. If *E* + *B*(*n*+1)^2^log⁡⁡*q*_*i*_ < ⌊*q*_*i*_/2⌋/2, we have *m* ← **F** **H** **E**.**D** **e** **c**(**s**_*i*−1_, **c**_1_); namely, **c**_1_ can be correctly decrypted.

**Proof**

By Lemma 7

(23) 〈c1,si−1〉=〈BitDecomp(c),e1〉+〈c,si′〉(mod⁡ qi)=B(n+1)2·⌈log⁡qi⌉+〈c,si′〉(mod⁡ qi)=B(n+1)2·⌈log⁡qi⌉+E+⌊qi2⌋·m (mod⁡ qi),

where **e**_1_ ← *χ* ^*N*^ and *N* = (*n*+1)^2^ · ⌈log⁡⁡*q*_*i*_⌉. If *E* + *B*(*n*+1)^2^log⁡⁡*q*_*i*_ < ⌊*q*_*i*_/2⌋/2, we have *m* ← **F** **H** **E**.**D** **e** **c**(**s**_*i*−1_, **c**_1_); namely, **c**_1_ can be correctly decrypted.

**Lemma 15 .**

Let **c**_1_ be a ciphertext of dimension *n* + 1 under **s**_*i*−1_ for *q*_*i*_, where 〈**c**_1_, **s**_*i*_〉 = ⌊*q*_*i*_/2⌋ · *m*_1_ + *e*_1_(mod⁡ *q*_*i*_) with |*e*_1_ | ⩽*E* < ⌊*q*_*i*_/2⌋/2. Let **c**_2_ ← **S** **c** **a** **l** **e**(**c**_1_, *q*_*i*_, *q*_*i*−1_, 2), The noise magnitude of **c**_2_ is at most (*q*_*i*−1_/*q*_*i*_) · *E* + (*n* + 1) · *B* + 1/2. If (*q*_*i*−1_/*q*_*i*_) · *E* + (*n* + 1) · *B* + 1/2 < ⌊*q*_*i*−1_/2⌋/2, we have *m* ← **F** **H** **E**.**D** **e** **c**(**s**_*i*−1_, **c**_2_); that is, **c**_2_ can be correctly decrypted.

**Proof**

By Corollary 10

(24) |E′|<(qi−1qi)·|E|+||si−1||1+12<(qi−1qi)·E+(n+1)·B+12.

If (*q*_*i*−1_/*q*_*i*_) · *E* + (*n* + 1) · *B* + 1/2 < ⌊*q*_*i*−1_/2⌋/2, we have *m* ← **F** **H** **E**.**D** **e** **c**(**s**_*i*−1_, **c**_2_).

## 8. Security and Parameters Settings

For a FHE scheme using modulus switching, it is most important to set up a reasonable ladder of decreasing modulus. The size of modulus is related to the dimension *n* of the LWE problem and the circuit depth *L*. Furthermore, the underlying security parameter *λ* is related to the dimension *n* of the LWE problem. However, it does not provide the concrete connection between the underlying security parameter and the dimension of the LWE problem in Regev's paper, nor the concrete parameters setting on its encryption scheme. It also does not provide the concrete method to set a concrete ladder of decreasing modulus based on a concrete security level and other parameters in the BGV scheme, even though BGV scheme is the first FHE scheme using modulus switching.

In this section, we will analyze the function between the lower bound in the dimension *n* of the LWE problem and the security level. Then we will give the method how to set the concrete ladder of decreasing modulus based on a certain security level and other parameters in our scheme.

### 8.1. The Dimension of the LWE Problem and the Security Level

In order to estimate the hardness of LWE for a concert set of parameters, we first consider the distinguishing attack LWE; namely, the adversary distinguishes (with some noticeable advantage) an LWE instance from uniformly random, which can result in that the semantic security of an LWE-based cryptosystem is to be broken with the same advantage. Given a point **b** that is either LWE instance or uniformly random. In order to do this attack, the adversary needs to find a short nonzero integral vector **v** such that **A** **v** = 0 mod *q*; namely, **v** is a short vector in Λ_*q*_^⊥^(**A** ^*t*^). Since Λ_*q*_^⊥^(**A** ^*t*^) = *q* · Λ_*q*_(**A** ^*t*^)*, we have **v** = *q* · **y**, where **y** is a short vector in the dual of the lattice Λ_*q*_^⊥^(**A** ^*t*^). Then the adversary tries to test whether the inner product 〈**v**, **b**〉 is close to zero modulo *q*. When **b** is a uniformly random instance, the test accepts with the probability exactly 1/2. When **b** = **A** ^*t*^ **s** + **e**, where **e** is sampled from a Gaussian distribution with standard deviation *σ*, we have 〈**v**, **b**〉 = 〈**v**, **A** ^*t*^ **s**〉 + 〈**v**, **e**〉 = 〈*q* · **y**, **A** ^*t*^ **s**〉 + 〈**v**, **e**〉 = 〈**v**, **e**〉 mod *q*, which is essentially Gaussian with standard deviation ||**v**|| · *σ*. When ||**v**|| · *σ* is not much larger than *q*, the adversary can distinguish the Gaussian from the uniform with advantage of being very close to exp⁡⁡(−*π* · (||**v**||·*σ*/*q*)^2^). In general, in order to do the distinguishing attack with high confidence, one needs ||**v**|| ⩽ *q*/(2*σ*), which need to reduce the basis well enough such that the shortest vector is of size roughly *q*/*σ*. We assume that the security depends on the ratio *q*/*σ*. Furthermore, we assume that the adversary will spend all the attack running time doing lattice reduction according to the paper [26].

The key point is to compute inner product 〈**v**, **e**〉 modulo *q* for a enough short vector in the distinguishing attack described above, which do not use the secret **s** of LWE sample. It means that the distinguishing attack still work whether the secret **s** is sampled from a Gaussian distribution or uniform. Next, we analyze the relation between the dimension of LWE and the security level.

A short vector used in the distinguishing attack can be got from lattice reduction algorithm. From the analysis of lattice reduction algorithms by Gama and Nguyen [27], the Hermite factor is regarded as the dominant parameter in the runtime of the reduction and the quality of the reduced basis. A reduced basis **B**(||**b**_1_|| ⩽ ||**b**_2_||,…, ⩽||**b**_*m*_||) of an *m*-dimensional lattice Λ has the Hermite factor *δ* ^*m*^ for *δ* ≥ 1 if ||**b**_1_|| = *δ* ^*m*^ · det⁡⁡(Λ)^1/*m*^. The term *δ* is called a quality parameter. In addition, Lindner and Peikert perform the experiments in the paper [26], which predict the runtime required to achieve a given root-Hermite factor *δ* in random *q*-ary lattices arising from LWE. The result of their experiments show that the logarithm of the runtime should grow roughly linearly in 1/log⁡⁡(*δ*). In particular, for a random *q*-ary lattices arising from LWE, the time (in seconds) that is spent to compute a reduced basis of quality *δ* is conservatively estimated at least as follows:

(25) log⁡⁡(time)⩾1.8log⁡⁡(δ)−110.

We note that the runtime estimated in (25) can be also applied in here to analyze our scheme. First, the random *q*-ary lattices for experiments in the paper [26] include the random *q*-ary lattices arising from LWE where the secret was sampled from a Gaussian distribution. Second, the encryption scheme described in the paper [26] is also based on the same LWE problem like our scheme; namely, the secret is choose from a Gaussian distribution.

Recall that the basis is required to be reduced well enough such that the shortest vector is of size roughly *q*/*σ* in the distinguishing attack. Thus the adversary needs to reduce the basis enough so that ||**b**_1_|| = *q*/*σ*. Moreover, for a random *q*-ary lattice of rank *n*, the determinant is *q* ^*n*^ with high probability. By the definition of quality parameter *δ*, a basis **B** that has quality parameter *δ* has ||**b**_1_|| = *δ* ^*m*^ · det⁡⁡(Λ)^1/*m*^ = *δ* ^*m*^ · *q* ^*n*/*m*^. From the result in paper [28], when lattice reduction algorithms is applied to Λ^⊥^(**A** ^*t*^), the shortest vectors are produced when *m* = (*n* · log⁡⁡*q*/log⁡⁡*δ*)^1/2^. For simplicity, we take *σ* = 1 such that ||**b**_1_|| = *q*/*σ* = *q*, then we have

(26) log⁡⁡q=log⁡⁡(δm·qn/m)=m·log⁡⁡δ+(nm)log⁡⁡q=2(n·log⁡⁡qlog⁡⁡δ)1/2.

We can solve for *n* and plug Equation (25) into it, then get *n* = log⁡⁡*q* · (log⁡⁡(time) + 110)/7.2 which is a function between *n* and *q*/*σ* (recall *q* = *q*/*σ*). In order to ensure the time that is spent to reduce the basis at least 10^*k*^, we need to set *n* to be at least

(27) n⩾(log⁡⁡(q/σ)·(k+110))7.2.

We thus obtain the relation between the dimension of LWE and the security level. If we want to get 80 bit security level we need to set *n*⩾log⁡⁡(*q*/*σ*) · 26.4, for 128 bit security level we need to set *n*⩾log⁡⁡(*q*/*σ*) · 33.1.

### 8.2. Setting Concrete Parameters

Based on our scheme, we first set a concrete ladder of decreasing modulus. For a certain security level, we recommend specific dimension and modulus values for a specific circuit level *L*.

#### 8.2.1. The Upper Bound of Noise

In order to obtain a suitable modulus, we need to find a common upper bound of noise for each circuit level.

Assume that we have a common upper bound *E* on noise magnitude, which means that the noise magnitude is at most *E* for all ciphertexts in all levels. Let **c**_1_ and **c**_2_ be two ciphertexts at level *i*. The noise magnitude is at most 12*nB* *E* after multiplication by following Lemma 11. Then, we apply the key switching, and the noise magnitude is at most 12*nB* *E* + *B*(*n*+1)^2^log⁡⁡*q*_*i*_ by following Lemma 12. Finally, we apply modulus switching, and the noise magnitude in this stage is at most

(28) (qi−1qi)·(12nB E+B(n+1)2log⁡⁡qi)+(n+1)·B+12.

According to our assumption, the above equation is less than *E*. The dominant term is 12*nB* *E*; thus, we have

(29) (qi−1qi)·12nB E<E2,

(30) (qi−1qi)·(B(n+1)2log⁡⁡qi)+(n+1)·B+12<E2.

We get *q*_*i*−1_/*q*_*i*_ < 1/(24*nB*) from Inequality (29), and we plug it into Inequality (30); then we have

(31) (124nB)·(B(n+1)2log⁡⁡qi)+(n+1)·B+12 ≈(124)·(n+1)·log⁡⁡qi+(n+1)·B ≈2(n+1)·B <E2.

We thus set *E* ≈ 8(*n* + 1) · *B*, which is the approximate common upper bound. We also get the ratio of *q*_*i*−1_ and *q*_*i*_ that is approximately 1/(24*nB*). Next we can set a concrete ladder of decreasing modulus.

#### 8.2.2. A Concrete Ladder of Decreasing Modulus

We first consider the smallest modulus. At the level 0, the noise magnitude is at most 12*nB* *E* after multiplication. In order for the correction of decryption to occur, we need to ensure 12*nB* *E* < *q*_0_/4. We can take *q*_0_ ≈ 48*nB* *E* ≈ 384*n* ^2^ *B* ^2^, which is approximately the smallest modulus.

Since *q*_*i*−1_/*q*_*i*_ ≈ 1/(24*nB*), we can derive *q*_1_,…, *q*_*L*−1_; for example, *q*_1_ = 24*nB* *q*_0_, *q*_2_ = (24*nB*)^2^ · *q*_0_,…, *q*_*L*−1_ = (24*nB*)^*L*−1^ · *q*_0_. We thus obtain a concrete ladder of decreasing modulus.

#### 8.2.3. The Concrete Parameters of Our Scheme

According to (27) and the largest modulus *q*_*L*−1_, we have *n*⩾(log⁡⁡(*q*_*L*−1_/*σ*) · (*k* + 110))/7.2, which is the lower bound of the dimension of LWE. We use the Gaussian parameter *σ* = 7 from the experiment in [28]. Since log⁡⁡(*q*_*L*−1_/*σ*) = log⁡⁡((384 · 24^*L*−1^ · (*nB*)^*L*+1^)/7) ≈ 8 + 5(*L* − 1) +(*L* + 1)(log⁡⁡*n* + log⁡⁡*B*), we have

(32) n⩾(3+5L+(L+1)(log⁡⁡n+log⁡⁡B))·(k+110)7.2.

*B* is the bound of Gaussian, and we use *B* = 2*n* from the statement in [20]. We then can obtain the lower bound of the dimension *n* from the circuit depth *L* as well as the security level.

For an 80-bit security level (*k* = 80) and different circuit depth *L*, we derive the parameters of our scheme, as shown in the Table 1.

### 8.3. Performance

The computational complexity of our scheme comes from homomorphic multiplication which includes three steps. The computational cost that computes the tensored ciphertext is $\tilde{O}(n^{2}\log^{2}q_{j})$. The computational cost in the step of key switching is $\tilde{O}(n^{3}\text{lo}{\text{g}}^{2}q_{j})$. The computational cost in the step of modulus switching is $\tilde{O}(n\log q_{j})$. As a result, the per-gate computation in our scheme is $\tilde{O}(n^{3}\text{lo}{\text{g}}^{2}q_{j})=\tilde{O}(n^{3}L^{2})$. As a comparison, in the Bra12 scheme the per-gate computation is $\tilde{O}(n^{3}\text{lo}{\text{g}}^{4}q_{j})$. This shows that our scheme is more efficient than the Bra12 scheme.

### 8.4. Bootstrapping

We also can use bootstrapping to achieve a leveled FHE scheme. Furthermore, by using bootstrapping, we can obtain a pure FHE scheme with an assumption of circular security. There is a detailed explanation about bootstrapping in paper [29].

In our scheme the depth of a decryption circuit is *O*(log⁡⁡*n* + log⁡⁡log⁡⁡*q*). We can regard the above leveled FHE scheme as a somewhat homomorphic encryption scheme. As long as we set the depth of circuit *L* > *O*(log⁡⁡*n* + log⁡⁡log⁡⁡*q*), our scheme is bootstrappable.

## 9. Conclusions

We have constructed a leveled FHE scheme using modulus switching based on the Bra12 scheme, and our scheme improves the efficiency of the Bra12 scheme. The per-gate computation in our scheme is $\tilde{O}(n^{3}\text{lo}{\text{g}}^{2}q_{j})=\tilde{O}(n^{3}L^{2})$, while it is $\tilde{O}(n^{3}\text{lo}{\text{g}}^{4}q_{j})$ in the Bra12 scheme. Furthermore, we have derived a function of the lower bound in the dimension *n* of the LWE problem and the security parameter. For an 80-bit security level and several different depth parameters, we have shown the concrete values of the dimension *n* of the LWE problem and the modulus *q* in each level. These concrete values for different parameters are very important in the fully homomorphic scheme that leverages modulus switching technique for noise management, which cannot be solved before.

## Figures and Tables

**Table 1:** The parameters of our scheme.

*L*	*n*	log⁡*q*_0_	log⁡*q*_*L*−1_
10	9400	63	149
20	19100	67	714
30	29200	70	1092
40	39500	72	1446
